# Disentangling prescribing behaviour of Cypriot physicians, within a complex framework of interacting

**DOI:** 10.1002/hpm.3480

**Published:** 2022-04-15

**Authors:** Mamas Theodorou, Antonis Kontemeniotis, Marios Kantaris, Antonis Farmakas

**Affiliations:** ^1^ Open University of Cyprus Latsia Cyprus; ^2^ Health and Social Services Research Centre American University of Cyprus Larnaca Cyprus; ^3^ University of Nicosia Nicosia Cyprus

**Keywords:** Cyprus, economic crisis, physicians prescribing behaviour

## Abstract

The purpose of the study is to investigate how physicians' prescribing behaviour in Cyprus adopts to the fragmented healthcare system and to the inadequacies of pharmaceutical market in times of economic crisis. A survey was carried out in using a postal questionnaire administered to a stratified sample of 320 physicians. The questionnaire used was the same with the one used in 2007 survey carried out in Greece and Cyprus, along with complementary questions for prescribing within economic crisis. The comparative analysis and assessment of the findings from the two surveys revealed that the current system and the inadequacies of pharmaceutical market in Cyprus expose physicians to a contrasting environment of public and private sector in terms of incentives, governance principles, financing and market structure. In contrast to public sector prescribers who have behaved in accordance with the governance principles, there is a strong motivation for private sector physicians to favour new branded products, and generally rejecting any ideas that could limit their clinical autonomy. Economic crisis seems to be unilaterally influential, as public sector physicians became more cost conscious while private sector prescribing is still resisting due to strong financial incentives.

## Highlights

- Prescribing behaviour of physicians in Cyprus amid economic crises.
- Pressures by patients, policy makers and pharma companies shape prescribing behaviour.
- Disentangling prescribing behaviour could lead to the revelation of opportunities for improvements.
- Differential prescribing could justify that decision‐making might be compromised for economic reasons.

## 1 BACKGROUND

Physician prescribing behaviour is influenced by many factors which are allowed to operate in the health care system according to its structure, governance, principles and incentives.^1^, ^2^, ^3^ Furthermore, the diversity on the type of the stakeholders involved in pharmaceutical market implies diversity of roles and objectives. In their everyday practice, physicians are exposed to pressures and expectations in the medication use process, namely patients, policy makers and pharmaceutical companies. A continued misalignment between these key players' goals could aggravate irrational prescribing, resulting in growth of expenditure and loss of health and quality of life for patients and society.^4^, ^5^ A recent survey revealed similarities in prescribing patterns among the southern European regions and a problem with irrational prescribing behaviour,^6^ while financial crisis has accelerated the enforcement of policies and measures to decrease pharmaceutical expenditure and promote rational prescribing.^7^

Cyprus, as a southern European country, shares similar problems and challenges, but also additional difficulties, due to its health system particularities, such as the lack of universal coverage, the large size of the private sector that is largely unregulated, the very high out‐of‐pocket payments and the long waiting lists that force public system beneficiaries to visit health providers in the private sector and bear the full cost. These particularities make the Cypriot health system quite different from the systems in other European countries, and its operating framework more complex, with conflicting interests and competing providers. However, there is lack of research about the factors that influence prescribing behaviour and the underlying reasons as to why the doctors prescribe irrationally. A valuable source of information is the survey conducted in 2007, aiming to investigate prescribing behaviour and its determinants amongst Greek and Cypriot primary care physicians. The questionnaire of this survey was designed to draw information on the criteria which justify prescription choice, the sources of physician information, the attitudes towards generic and new innovative drugs, and the importance of drug cost, adverse drug reactions and safety.^1^

However, this study is subject to limitations due to the structure of Cyprus healthcare system and the lack of universal coverage. The system is running for many years as a two‐tier healthcare system in which a highly regulated and over‐burdened publicly funded sector coexists with a privately financed sector with the minimum of control and regulation. The total health expenditure has been almost evenly distributed between the two sectors, and the share of private expenditure is one of the highest in the EU countries.^8^, ^9^

Inevitably, the structure, governance and incentives are different between the two sectors. In the private sector, patients are confronted with the prices as defined by the statutory pricing method applied to all marketed products and for which external price referencing is used for all imported pharmaceuticals.^10^ On the contrary, public sector drugs are procured centrally through international tenders which lead to lower prices than those set in the statutory price list, while restricting the bandwidth of therapeutic options by awarding the right to a single firm to supply the public sector for the particular category that is being tendered.^9^, ^10^ The current system incentivises the use of branded drugs in the private sector due to the lack of any public mechanism to monitor and control the prescribing behaviour of physicians. Provider induced demand for on‐patent expensive product is further enabled by the remuneration system for pharmacists in the private sector as they are paid on a flat percentage within a broadly defined price band.^9^, ^10^ The available pharmaceutical products in the public sector accounts for one third of all marketed products and are listed on a positive formulary, which demands to stick to prescription protocols restrictions and guidelines managing the prescribing practices of doctors. The majority of formulary restrictions mandate the use of generics or the cheapest product as first choice.^9^, ^10^

Additionally, Cyprus suffers from what is known as the ‘small market’ problem, facing significant limitations in terms of medicine availability. The lower number of products compared to other member state markets might undermine competitiveness, leading to inelastic pricing and/or restrict significantly the bandwidth of pharmaceutical therapeutic options.^9^, ^10^

Consequently, any results concerning the factors influencing physician prescribing should be cautiously evaluated, given the different environment between the public and private sector and the market limitations. In this context, it would be interesting to explore how the economic crisis affected prescribing behaviour as well. The recent economic crisis along with the agreed memorandum of understanding with creditors, led to stringent austerity measures that affected decisions about a whole range of public healthcare services including pharmaceuticals.

Therefore, within this new economic and social environment, we carried out a new survey in 2015 using the same questionnaire with the one used in 2007 survey, along with complementary questions for prescribing amid economic crisis, in order to investigate whether the health system structure and the temporal changes in Cyprus economy and pharmaceutical market, affected the way the main determinants of prescribing operate.

## 2 METHODS

The questionnaire used consists of seven sections, the six of which are identical to those included in the questionnaire of the 2007 survey, developed and validated by the Department of Health Economics at the National School of Public Health in Greece. This approach allow us to outline in a comparative way the findings of the Cypriot physicians in 2007 and 2015, aiming to investigate the factors influencing prescribing and possible changes between the two dates. The seventh section included questions concerning prescribing within economic crisis referring only to the year 2015. Overall, the “new” questionnaire had 45 semi‐closed questions, and was structured around the following topics: characteristics of physicians who participated, determinants of physician prescribing behaviour and their main sources of information, opinion about the cost of pharmaceuticals to the patient, attitudes towards the prescription of generics, new pharmaceutical products and safety issues, and finally prescribing within economic crisis.

This questionnaire was piloted to a group of40 physicians, allowing to some minor changes, especially for the better understanding of the questions in the seventh complementary section. In May 2015, the final questionnaire with a cover letter and a prepaid return envelope was mailed to a convenient sample of 320 doctors from all over‐Cyprus, stratified by region, specialty, sex and employment sector. Physicians not authorised to prescribe were excluded from the sample (interns and doctors in the specialties of radiology, nuclear medicine, microbiology, haematology, anaesthesiology and forensic medicine). Despite the proportional stratified sampling technique, the two samples (2007 and 2015) are not made up of the same doctors and this is a limitation of the research. This problem is somewhat limited due to the large sample size, accounting for approximately 9%, of the total number of active prescribing doctors in Cyprus.

In total, and after two reminders, 254 completed questionnaires were received (response rate 79.4%). Data collection was followed by quality control, codification, recording and statistical analysis with IBM SPSS, version 21.0. Comparisons of physicians' responses across sectors (private and public) and between the 2 years were done by cross tabulation against different attributes by performing a Chi‐square test at 95% confidence intervals.

## 3 RESULTS

As the six sections of the questionnaire used in our 2015 survey are identical to those in2007, the results of the two surveys are presented in tables in a comparative way.

### 3.1 Demographic characteristics

A relatively larger sample of physicians has participated in our study (254) compared to that of 2007 (193), with similar experience patterns in terms of practicing years. Educational and training activities remain at similar levels with the exception of the continuous education element, which is lower in 2015 (41.3% vs. 56.5%). In terms of specialties, the top six specialties remain the same representing the 77.6% (2015) and 64.2% (2007) of the participants, while significantly higher rates are recorded in 2015 compared to 2007 concerning physicians' computer acquaintance and use of Internet (High/Satisfactory Use 84% vs. 70%).

### 3.2 Influential factors and information sources

Clinical effectiveness is predominantly selected (by 95%) as the basic criterion when prescribing. Similar findings were recorded in the survey of 2007, with no statistically significant difference between the two.

“Medical congresses”, “sales representatives/scientific collaborators of pharmaceutical companies” and “medical journals” are mainly preferred by the physicians (>50%) to justify their prescription choices and to a lesser extend “medical textbooks”, “medical libraries and Internet databases”. Worth mentioning is the high reliance of Cypriot physicians on pharmaceutical representatives in both surveys (58.3% in 2015% and 61.1% in 2007), significantly higher than that for Greek physicians (51.9%). In general, there were no temporal changes in the rank order list of the information sources. However, there were significant differences between public and private sector physicians concerning their preference for medical congresses (2015: 56.3% vs. 70.3% *p* 0.012, 2007: 61.8% vs. 74.6% *p* 0.064) and sales representatives/scientific collaborators of pharmaceutical companies (2015: 52.6% vs. 63%, *p* 0.068, 2007: 41.2% vs. 73%, *p* ≤ 0.001) in both years.

The influence of pharmaceutical representatives is further supported by the finding that more than 40% in both surveys consider that sales representatives/scientific collaborators of pharmaceutical companies can affect “a lot” or “enough” their prescribing. It is interesting to note that the significantly lower importance ascribed to the role of pharmaceutical representatives by the public sector physicians compared to the private sector in 2007, has increased in 2015 leading to insignificant differences between the two sectors (Table 1).

**TABLE 1:** Cross sector comparison on prescribing behaviour of physicians

To what extend the information provided by the pharmaceutical sales representative influence physician prescribing				
Year	Sector	Very often/Often	Rarely/Hardly ever	
2015	Private	44.8%	55.20%	
	Public	42.2%	57.80%	
2007	Private	55.6%	44.40%	*p* = 0.000
	Public	28.4%	71.60%	

**How frequently do physician consult their colleagues on good prescribing practice**				
Year	Sector	Very often/Often	Rarely/Hardly ever	
2015	Private	21.8%	78.2%	*p* = 0.002
	Public	37.8%	62.2%	
2007	Private	15.3%	84.7%	*p* = 0.000
	Public	42.7%	57.4%	

**Frequency of changing initial prescribing**				
Year	Sector	Very often/Often	Rarely/Hardly ever	
2015	Private	6.7%	93.3%	*p* = 0.002
	Public	18.5%	81.5%	
2007	Private	13.1%	86.9%	
	Public	16.2%	83.8%	

**How frequently do patient put the physician under pressure to prescribe a new drug?**				
Year	Sector	Very often/Often	Rarely/Hardly ever	
2015	Private	20.6%	79.4%	*p* = 0.071
	Public	29.6%	70.4%	
2007	Private	4.1%	95.9%	*p* = 0.000
	Public	19.7%	80.3%	

Consulting colleagues (“very often” or “often”) in order to be kept abreast on good prescribing practice is observed in the one third of the physicians. As outlined in Table 1 there are significant differences across sectors in both years. Public sector physicians are consulting their colleagues more frequently compared to the physicians in the private sector.

Physicians appeared to change their initial prescribing choice “often” or “very often” at a rate of 12% and 14% in 2015 and 2007 respectively. Public sector physicians change their initial choice more frequently, especially in 2015 where the gap between the two sectors has increased (Table 1). The importance of each reason for changing initial prescribing remains relatively constant when comparing the 2 years. Safety issues are predominantly the most important reason. However, there are differences across the two sectors. The circulation of a new drug is significantly more important for the private sector physicians (39.4% vs. 16.3%, *p* ≤ 0.001) while side effects are more important for the public sector physicians (97.8% vs. 86.7%, *p* ≤ 0.001).

With regard to the patients' pressure as perceived by physicians, there is a remarkable increase of physicians reporting patients demanding a new drug to be prescribed in 2015, compared to 2007. In particular, 24.7% of the physicians “often” or “very often” were put under pressure to prescribe a new drug, compared to 10% in 2007. As illustrated in Table 1, public sector physicians are more frequently exposed to pressure regarding prescribing decisions. However, temporal changes are more profound in private sector, resulting in reduction of the gap between the two sectors in 2015.

### 3.3 The role of cost in prescribing

Regarding the cost and its role in prescribing, the findings indicate that cost not only plays a significant role, but it gained more attention in 2015. In particular, 74% of the physicians in 2015 consider cost “*important*” or “*highly important*” compared to the 63.1% in 2007 (*p* ≤ 0.001). There are no significant differences across sectors in both years.

The same picture applies in relation to patients complains about cost of drug therapy. In particular, patients were reported to complain more frequently in 2015 compared to 2007 (58.3% vs. 43.23%, *p* = 0.001), with the highest changes being recorded in public sector. In particular, in 2015%, 60.7% of public sector physicians reported that patients complain “*often*” or “*very often*” about cost of drug therapy, while in 2007 the figure was 35.8%.

### 3.4 Generic drug prescribing

Physicians' attitude towards generics is rather positive based on 2015 survey data (Table 2). More specifically, they find their quality, safety and effectiveness “excellent” or “satisfactory” at 61.7%, 70% and 65.3% respectively. In addition, INN prescribing, namely prescribing based on drug substance, is considered “feasible” or “very feasible” by 62.3% of physicians. This positive view appears to be in line with the reported frequency (73.7%) of generic prescribing. No statistically significant changes were found when comparing 2007–2015. On the contrary, the differences between the public and private sectors in the 2015 study are statistically significant.

**TABLE 2:** Physicians attitudes towards generic prescribing (2015)

	Response	Private (%)	Public (%)	*p*value	Private + Public (%)
Quality of generic	Excellent/Satisfactory	69.1	52.6	0.003	61.7
Safety of generic	Excellent/Satisfactory	72.7	66.7	0.254	70.0
Effectiveness of generic	Excellent/Satisfactory	71.5	57.8	0.013	65.3
Feasibility of INN prescribing	Feasible/Very feasible	52.1	74.8	0.000	62.3
Frequency of generic prescribing	Often/Very often	69.7	78.5	0.084	73.7

### 3.5 New drug prescribing and formulary abolishment

Attitudes towards new drugs with respect to the price remain similar in both years. In our survey, 58.3% of physicians believe that “*a higher price does not necessarily imply better outcome*”, which approximates the corresponding rate of 2007 (63.3%). Interestingly, despite that physicians in 2015 have downgraded significantly their perceptions of new drug effectiveness, since only 14% consider new drugs “*clearly more effective*” compared to 24.4% in 2007 (*p* = 0.002), private sector physicians' perception was significantly more positive compared to their public sector colleagues in both years (2015 *p* = 0.011,2007 *p* = 0.034).

With respect to formulary based prescribing, 34.3% in 2015 consider that public sector formulary abolishment “*would make patient better off* ” and 10.7% “*would accelerated new product launching*”. The rest of the physicians consider that this “*would either increase health expenditure*” or “*would be in favour of pharmaceutical companies*”. Considering the last viewpoints as negative consequences and the first two as positive, there is a significant difference between public and private sector physicians with respect to their opinion. Private sector doctors have a more positive opinion for formulary abolishment compared to their colleagues in the public sector.

### 3.6 Adverse drug reactions (ADRs) and safety

Not much has been changed since 2007 concerning the sources that physicians consult to get informed about ADRs. The National Drug Agency, the Internet, the pharmaceutical companies and to a lesser extend the colleagues, and the media are the major sources reported. However a significant increase of Internet's role is observed in 2015 (50% vs. 37.8% *p* = 0.008). Across sectors a higher preference is reported for drug representatives in the private sector (44.2% vs. 25.9%, *p* ≤ 0.001).

Side effects appear to be a major cause of prescription choice modification. More than 97% of doctors report that in case of side effects their prescription pattern changes, while the corresponding figure in 2007 was 90.2% (*p* = 0.0007). Nevertheless, the majority of doctors do not inform the authorities about side effects despite that a significant improvement is noted in reporting ADRs to authorities in 2015 (36.1% vs. 11.9%, *p* ≤ 0.001).

### 3.7 Prescribing within economic crisis

Table 3 presents the results from the last section of the 2015 survey based on complementary information concerning specific issues related to the economic crisis.

**TABLE 3:** Prescribing within economic crisis

	Private sector (%)	Public sector (%)	Private + Public (%)
**How frequently do patient put you under pressure to prescribe a cheaper drug**			
Not at all	26.1%	19.3%	23.0%
A little	31.5%	40.0%	35.3%
Average	25.5%	23.7%	24.7%
Enough	16.4%	14.1%	15.3%
A lot	0.6%	3.0%	1.7%
	*p* = 0.212		
**Did you notice any change on prescribing following the implementation of fees for each prescribed item in the public sector from Aug 2013?**			
Not at all	54.7%	20.0%	38.9%
A little	26.7%	33.3%	29.7%
Average	10.6%	20.7%	15.2%
Enough	7.5%	20.0%	13.2%
A lot	0.6%	5.9%	3.0%
	*p* = 0.00		
**Which me‐too product would you prescribe for a beneficiary?**			
Product available in private sector	50.9%	14.1%	34.3%
Product available in public sector	15.2%	51.1%	31.3%
Any of those	33.9%	34.8%	34.3%
	*p* = 0.00		
**Among products with the same active ingredients, which one would you prescribe for a beneficiary?**			
Product available in private sector	32.7%	10.4%	22.7%
Product available in public sector	22.4%	57.0%	38.0%
Any of those	44.9%	32.6%	39.3%
	*p* = 0.00		
As a **private sector physician** in what cases do you prescribe a **product available in the public sector**			
Product not available in the private sector			81.8%
Patient's request			48.5%
In need for cheaper product			38.2%
Public sector physician request			18.8%
As a **public sector physician** in what cases do you prescribe a **product available in the private sector**			
Not available in the public sector			85.9%
Inaccessible products due to restrictions			46.7%
Patient's request			39.3%
Following treatment failure with the available products			37.0%
Perception of superior products in the private sector			19.2%
To avoid periodic changes in drug treatment due to changes in tenders			12.6%
Private sector physician request			9.6%

Despite economic crisis, only 17% of the physicians were put under pressure (“*enough*” or “*a lot*”) to prescribe a cheaper drug. No statistically significant differences found across sectors.

The introduction of user charges for each prescribed product since August 2013 caused changes (“*enough*” or “*a lot*”) in prescribing, significantly higher in the public sector, as expected (25.6% vs. 8.1%, *p* ≤ 0.001).

As a proxy variable for cost saving, the following cases where investigated: the attitudes towards me‐too drugs and products with the same active ingredient when prescribing to a beneficiary.

Differential prescribing to beneficiaries across sectors was evident, based on physicians' attitudes towards me‐too drugs with the same active ingredient. In both cases there is a significantly different attitude in prescribing, between physicians. In the case of me‐too drugs over 50% of private sector physicians prefer to prescribe a product available in the private sector. Similarly, over 50% of public sector physicians prefer the available products in the public sector. For products with the same active ingredients, the private sector physicians are rather indifferent between the available products in the two sectors at a rate of 44.9%. On the other hand, 57% of the public sector physicians prefers to prescribe the predominantly available generics in the public sector.

Two set of questions were formed in order to understand the reasons for cross‐sector prescribing. The corresponding results in Table 3 reveal that the two sectors are complementing each other in terms of product availability, namely none of them is adequate to meet the demand by its own. In the public sector there are issues with accessibility since 46.7% of doctors seek products in the private sector due to prescribing restrictions. In the private sector there is an issue with affordability since 38.2% of physicians are in need to offer a less costly drug treatment.

## 4 DISCUSSION

Doctor prescribing behaviour has been extensively studied resulting in many different influential factors, which can interact in unpredictable way.^10^ In an effort to disentangle this complex decision making process we should bear in mind that physician's knowledge and objectives as the basic elements for prescribing, are amenable to adaptations conditional to the pressure exercised by patients, policy makers and pharmaceutical companies, and motivated by the existing structure of incentives. The boundaries of these adaptations are set by the healthcare system and the pharmaceutical market. Τhe analysis that follows through the abovementioned framework could shed light on how doctors in Cyprus prescribe.

The most highly valued variable found to be the “*proven clinical effectiveness*” which is in line with the findings of many other relevant studies.^1^, ^2^, ^11^, ^12^ The term effectiveness reflects the primary concern of the physicians to maximize therapeutic result. This underlines the fact that prescribing is a knowledge‐intensive process. The conception of knowledge needs and how these are sought to be met is a product of interaction of the prescriber with the above mentioned key actors, each one of which can potentially provide different types of information.^13^, ^14^

According to our findings, the drug representatives are considered a credible and influential information source. The medical congresses, for which the private sector physicians revealed a stronger preference, could indirectly reflect the interference of the drug representatives since one of the most frequently used marketing tool is the “all‐expenses‐paid trips to conferences”. According to Wadmann and Bang, drug representative operates not as conveyors of information but as those who contribute by helping physicians to organise this information, given the time constraints that the physicians are confronted with.^13^

The relatively higher reliance of the private sector physicians on drug representatives as a source of information could be due to the greater opportunities for the promotion of branded drugs. This is in line with knowledge needs of the private sector physicians, who are predominantly centred on the branded drugs, which can be differentiated from those in the public sector and be perceived by the patients as a quality signal.

Τhe public sector physicians οn the other hand, acquire knowledge through the practice or the opinion of their hospital colleagues. This‐type of knowledge is highly valued and considered an influential source of information for primary care physicians.^16^

The contrasting environment of the two sectors in terms of incentives, governance principles, financing and market structure is highly reflected in our findings. Given the strong motivation of the private sector physicians to strive for quality, it is not surprising that their perception of new drug effectiveness was significantly more positive compared to that of their colleagues in the public sector. Additionally, they were more frequently willing to change their initial prescribing due to the circulation of a new drug, and more ready to defend their clinical autonomy by opposing to the idea of formulary based prescribing.

The resistance to INN prescribing despite the “highly rated reputation” of generics by the private sector physicians, could be attributed to the strong incentives favouring branded products.^15^, ^16^ This contrast was also observed for Greek physicians who, despite their positive view on generics, they prefer to prescribe the branded products.^17^, ^18^, ^19^ On the other hand, the high rates of generic prescribing by public sector physicians is not solely due to their favourable attitude towards generics but to some extend is imposed by policy makers.

Investigating the importance of cost in prescribing, it appears significantly increased. The frequency of patients' complaints about the cost of therapy increased significantly in 2015 compared to 2007 (60.7% vs. 35.8%), especially from public system beneficiaries. This could be attributed partly to the introduction of fees for each prescribed item from August 2013, despite that a recent study concluded that the introduction of these charges did not cause “*great dissatisfaction or distrust to the public*”.^20^ The increased frequency of complaints for cost can also be attributed to the fact that the majority of beneficiaries tend to use, regularly or occasionally, private healthcare services and bear cost by out‐of‐pocket (OOP) payments. As the beneficiaries mainly belong to the middle to low income groups, those exposed to OOP payments would be more vulnerable to the impact of the economic crisis.

Further to the economic crisis, the temporal changes in pharmaceutical market appear to have an impact on physicians' prescribing behaviour. Namely, the increasing imbalance between private and public market with respect to the number of available products could potentially enhance the use of private sector market. In particular, from year 2007 to 2015 pharmaceutical products have increased in the private market by 160% while the public sector market remained relatively constant.^21^ This imbalanced availability is reflected in the results through the higher exposure of public sector physicians to pressure from patients to prescribe a new drug and the higher frequency reported for changing their initial prescribing.

It becomes clear that physicians' decision‐making in prescribing adapts to the major deficiencies of the divided healthcare system in public and private, and the imperfections of pharmaceutical market in Cyprus. Consequently, there is a strong motivation for private sector prescribers to favor new branded products, and rejecting any ideas that could limit their clinical autonomy. In contrast, public sector prescribers have to behave in accordance to the governance principles and operate within limited sources despite having more opportunities to acquire valued knowledge. Economic crisis seems to be unilaterally influential, as public sector physicians became more cost conscious while private sector prescribing is still resisting due to strong financial incentives.

This study shares similar limitations to the 2007 study given the commonalities in the methodology for data extraction.^1^ However, the use of identical questionnaires and methodologies allowed direct comparison with the corresponding results in 2007 adding value to our analysis by allowing temporal changes to be captured. As with any survey, the results should be cautiously interpreted given the unavoidable contrast between attitude and action.

## 5 CONCLUSION AND POLICY IMPLICATIONS

The fragmented healthcare system and the inadequacies of pharmaceutical market expose Cypriot physicians to a contrasting environment in terms of incentives, governance principles, financing and market structure. Prescribing behaviour is further shaped by the pressure exercised by patients, policy makers and pharmaceutical companies.

Based on the results, notable differences were recorded in the physicians' prescribing behaviour between the private and public sector, routed mainly in the complexity of health environment, created by the previously mentioned different and conflicting factors. Consequently any intervention made to reverse prescribing patterns should take into consideration all attributes in order to be influential. Any measures should take into account the attitudes and perceptions of doctors. Understanding and decoding doctors' prescribing behaviour and aligning accordingly the incentives in place for all actors, are prerequisites for the appropriate measures, which not only will affect efficiency but also can prevent potential conflicts between health providers, stakeholders and the policy makers.

It should be noted that the findings of the present study and those of 2007 are no longer valid since the introduction of the new healthcare system of universal coverage from 1st June 2019, has completely changed the health environment in Cyprus (For a very brief description of the new integrated healthcare system in Cyprus and the ongoing implementation process, see the link https://eurohealthobservatory.who.int/countries/cyprus/). The Ministry of Health and the Health Insurance Organization should inform and “train” physicians as well as patients, to control over‐prescribing and polypharmacy. In addition, therapeutic protocols should be introduced in conjunction with increased penetration of generics by addressing the belief that generic medicines are of low therapeutic value; This can happen through continuous information to both physicians and patients. Perhaps the time is one of the most appropriate for measures since the new healthcare environment is more receptive to significant changes.

Finally, disentangling prescribing behaviour through this complex framework could effectively lead to the revelation of the opportunities for improvements. However, further analysis is warranted in order to realize the magnitude of this influence and to what extend this behaviour is related to prescribing practice that could undermine further the healthcare system of Cyprus in terms of financial protection, efficiency in allocation of resources and coordination among providers.^22^ The results of such surveys can facilitate decision‐makers towards the planning and implementing measures and policies in the pharmaceutical market in Cyprus.

## CONFLICT OF INTEREST

The authors declare that they have no competing interests.

## ETHICS STATEMENT

Not applicable.

## AUTHORS CONTRIBUTIONS

MT, MK and AF designed the study, coordinated the data collection, and reviewed the manuscript. AK was responsible for the statistical analysis and wrote the first draft of the paper.

## Data Availability

The data that support the findings of this study are available on request from the corresponding author. The data are not publicly available due to privacy or ethical restrictions.

## References

[1] Theodorou M , Tsiantou V , Pavlakis A , et al. Factors influencing prescribing behaviour of physicians in Greece and Cyprus: results from a questionnaire based survey. BMC Health Serv Res. 2009;9:150. 19695079 10.1186/1472-6963-9-150 PMC2737540

[2] Schumock GT , Walton SM , Park HY , et al. Factors that influence prescribing decisions. Ann Pharmacother. 2004;38(4):557‐562. 14966259 10.1345/aph.1D390

[3] Custers T , Hurley J , Klazinga NS , Brown AD . Selecting effective incentive structures in health care: a decision framework to support health care purchasers in finding the right incentives to drive performance. BMC Health Serv Res. 2008;8. 10.1186/1472-6963-8-66 PMC2329630 18371198

[4] Maynard Α , Bloor K . Dilemmas in the regulation of the market for pharmaceuticals. Health Aff. 2003;22:31‐41. 10.1377/hlthaff.22.3.31 12757269

[5] Mossialos E . European Observatory on Health System and Policies Series.Regulating pharmaceuticals in Europe: striving for efficiency, equity, and quality.

[6] Tsiantou V , Moschandreas J , Bertsias A , et al. General Practitioners’ intention to prescribe and prescribing patterns in selected European settings: the OTCSOCIOMED project. Health Pol. 2015;119(9):1265‐1274. 10.1016/j.healthpol.2015.06.006 26188356

[7] Vogler S , Zimmermann N , Leopold C , de Joncheere K . Pharmaceutical policies in European countries in response to the global financial crisis. Southern Med Review. 2011;4(2):69‐79. 23093885 10.5655/smr.v4i2.1004 PMC3471176

[8] Theodorou M , Charalambous C , Petrou C , Cylus J . and World Health Organization . Cyprus health system review: health system in transition. 2012;14(6):1‐128. 23149260

[9] Kanavos P , Wouters O . Transitioning to a national health system in Cyprus: a stakeholder analysis of pharmaceutical policy reform. Bull World Health Organ. 2015;93:606‐613. 26478624 10.2471/BLT.14.148742 PMC4581641

[10] Buusman A , Andersen M , Merrild C , Elverdam B . Factors influencing GPs’ choice between drugs in a therapeutic drug group. A qualitative study. Scand J Prim Health Care. 2007;25:208‐213. 18041657 10.1080/02813430701652036 PMC3379761

[11] Pinto J , Ferreira da Silva A , Curto J . Determinant values in the medical act of prescribing in the Portuguese context. Journal of Medical Marketing. 2010;10:213‐230.

[12] Karampli Ε , Triga Ε , Kyriopoulos J , Athanasakis K , Tsiantou V . Views of physicians and patients with chronic conditions on generic medicines in Greece after the introduction of measures to promote their consumption: findings from a qualitative study. Generics Biosimilars Initiative J. 2016;5(1):9‐20. 10.5639/gabij.2016.0501.005

[13] Wadmann S , Bang LE . Rationalising prescribing: evidence, marketing and practice‐relevant knowledge. Soc Sci Med. 2015;135:109e116 25965891 10.1016/j.socscimed.2015.04.032

[14] Prosser H , Walley T . New drug prescribing by hospital doctors: the nature and meaning of knowledge. Soc Sci Med. 2006;62:1565‐1578. 16199121 10.1016/j.socscimed.2005.08.035

[15] Granlund D . Are private physicians more likely to veto generic substitution of prescribed pharmaceuticals? Soc Sci Med. 2009;69:1643e1650. 19815322 10.1016/j.socscimed.2009.09.016

[16] Iizuka T . Physician agency and adoption of generic pharmaceuticals. Am Econ Rev. 2012;102:2826e2858. 29522299 10.1257/aer.102.6.2826

[17] Tsiantou V , Zavras D , Kousoulakou H , Geitona M , Kyriopoulos J . Generic medicines: Greek physicians' perceptions and prescribing practices. J Clin Pharm Therapeut. 2009;34(5):547‐554. 10.1111/j.1365-2710.2009.01037.x 19744010

[18] Vandoros S , Stargardt T . Reforms in the Greek pharmaceutical market during the financial crisis. Health Pol. 2013;109(1):1‐6. 10.1016/j.healthpol.2012.08.016 22959163

[19] Frouzi E , Chatzea VE , Sifaki Pistolla D , Saridi M , Rekleiti M , Souliotis Κ . Knowledge and attitudes of Greek Physicians towards generic prescribing after the economic crisis. Int J Pharma Sci Res. 2013;4:125‐133.

[20] Theodorou M . Testing the waters for GeSY: the opinion of patients for cost sharing arrangements in the public health care system in Cyprus. Cyprus Economic Policy Review. 2014;128(2):37‐59.

[21] Ministry of Health of Cyprus . Price Lists and Formularies for Pharmaceutical Products.

[22] 2014 IMF Paper: an Assessment of the Proposed National Health System in Cyprus. http://www.moh.gov.cy/MOH/MOH.nsf/page09_en/page09_en?OpenDocument

